# Biochemical recurrence after radical prostatectomy: what does it mean?

**DOI:** 10.1590/S1677-5538.IBJU.2016.0656

**Published:** 2018

**Authors:** Rafael Tourinho-Barbosa, Victor Srougi, Igor Nunes-Silva, Mohammed Baghdadi, Gregory Rembeyo, Sophie S. Eiffel, Eric Barret, Francois Rozet, Marc Galiano, Xavier Cathelineau, Rafael Sanchez-Salas

**Affiliations:** 1 Department of Urology, Institut Montsouris, Université Paris-Descartes, Paris, France; 2 Divisão de Urologia, Faculdade de Medicina ABC, São Paulo, Brasil; 3 Divisão de Urologia, Universidade de São Paulo, São Paulo, Brasil

**Keywords:** Prostatic Neoplasms, Prostate-Specific Antigen, Prostatectomy

## Abstract

**Background:**

Radical prostatectomy (RP) has been used as the main primary treatment for prostate cancer (PCa) for many years with excellent oncologic results. However, approximately 20-40% of those patients has failed to RP and presented biochemical recurrence (BCR). Prostatic specific antigen (PSA) has been the pivotal tool for recurrence diagnosis, but there is no consensus about the best PSA threshold to define BCR until this moment. The natural history of BCR after surgical procedure is highly variable, but it is important to distinguish biochemical and clinical recurrence and to find the correct timing to start multimodal treatment strategy. Also, it is important to understand the role of each clinical and pathological feature of prostate cancer in BCR, progression to metastatic disease and cancer specific mortality (CSM).

**Review design:**

A simple review was made in Medline for articles written in English language about biochemical recurrence after radical prostatectomy.

**Objective:**

To provide an updated assessment of BCR definition, its meaning, PCa natural history after BCR and the weight of each clinical/pathological feature and risk group classifications in BCR, metastatic disease and CSM.

## INTRODUCTION

Prostate cancer (PCa) is the most commom solid-organ male malignancy and second only to lung cancer in mortality in the United States (1). Radical prostatectomy (RP) remains the primary treatment for localized PCa and has been performed for many years with excellent oncologic control. However, approximately 20-40% of patients with clinically localized PCa will present biochemical recurrence (BCR) after RP (2-4).

Prostatic specific antigen (PSA) has been used for detection of recurrent disease for more than 35 years (5). Right after its introduction as a BCR marker, men with distinct diseases were treated similarly. Over time, it was possible to realize that PSA relapse has different meanings accordingly to clinicopathological features, as Gleason score (GS), PSA doubling time (PSA-DT), clinical stage and surgical margins status. Following this trend, individualized treatments emerged and authors proposed different PSA cut-off to define BCR, aiming to intervene in the best moment of PCa recurrence to achieve best perspectives of cure in each patient. In this way, several BCR criteria were adopted, impairing studies comparison and the practice of generalist doctors. Attempts to standardize BCR definition have been performed in last years, but there is no consensus at this moment (6, 7).

Despite lack of consensus, PSA remains the main tool to assess disease progression after RP. Conventional imaging appears to be useless for re-staging PCa after RP at an early enough stage, while new image techniques have been developed, mainly in nuclear medicine area (8). The natural history of BCR after surgical procedure is highly variable, but it is important to distinguish biochemical and clinical recurrence and to find the correct timing to start multimodal treatment strategy (9, 10). We reviewed the current medical literature to provide an updated assessment of BCR definition, its meaning, natural history and differences among risk groups.

## MATERIAL AND METHODS

A review was performed in July 2016 in Pubmed database, in English language, to identify studies about BCR after RP. Keywords comprised “biochemical recurrence”, “radical prostatectomy”, “definition”, “risk factors”, “nomograms” and “risk groups”. We selected 236 papers related to BCR after RP and most relevant ones were included in this review aiming to assess differences in BCR definition and the role of each clinical and pathological feature as a risk factor for biochemical and clinical relapse. Therapeutic management of BCR was not addressed in this review.

### Definition

Strict definition of BCR is important to identify patients at risk for disease progression and to enable comparisons among men submitted to CaP treatment in studies.

In 1994, the American Society for Therapeutic Radiology and Oncology (ASTRO) formed a committee to standardize definition of BCR after external-beam radiotherapy. It led to a Consensus Conference in 1996 and recommendation statement, last reviewed in 2005 (11).

Biochemical failure standardization after RP has been studied in more recent years. In the setting of organ confined disease, PSA is supposed to reach undetectable value after RP. However, undetectable value is dependent on the assay used in each institution, thus it is not uniform among the studies. Another consideration to be made is that patients get undetectable PSA level approximately 4 weeks after surgery, as serum half-life of PSA is about 3.2 days (12). A rising serum PSA level after achieving undetectable value is the first sign of reccurent disease.

Amling and colleagues analyzed the use of various PSA cut point definitions for determining biochemical progression after RP in 2001. They evaluated the percentage of patients with a continued PSA increase after each cut point to define the most appropriate PSA level to determine disease progression. Progression of PSA rates were 49%, 62% and 72% after reaching PSA levels of 0.2, 0.3 and 0.4ng/mL, respectively. They concluded PSA 0.4ng/mL or greater was the most appropriate cut point to define BCR (13).

In 2003, Freedland and colleagues performed a retrospective survey to determine the ideal cutpoint for defining BCR after RP. A series of cutpoints were examined (0.1-0.5ng/mL). They determined the ideal cutpoint as the lowest PSA level associated with a high risk of PSA progression in 1 years and 3 years. The risk of PSA progression in 1 year and 3 years among patients with postoperative PSA >0.1ng/mL was 36% and 67%, respectively. For PSA levels greater than 0.2ng/mL, these rates were 86% and 100%, respectively. Further increases in the threshold for failure did not increase progression rates. It led them to conclude that PSA greater than 0.2ng/mL would be an appropriate cutpoint to define PSA recurrence after RP (14).

Stephenson and colleagues evaluated metastatic disease progression as primary end-point to determine the best PSA cutpoint for BCR definition. In this multivariate regression analyzes performed in 2006, they concluded BCR defined as a PSA ≥0.4ng/mL and rising, best explains metastatic disease progression and was proposed as the standard for reporting outcomes after RP (15).

In a similar and recent study with a large cohort, Toussi and colleagues found a single PSA ≥0.4ng/mL had the strongest association between biochemical recurrence and sistemic progression. This PSA threshold was associated with PSA rising over 5 years in 74% of patients (6).

Recent studies have proposed to consider PSA value lesser than 0.2ng/mL for starting salvage therapy in high-risk patients. Mir and colleagues stratified patients into favorable and unfavorable groups and suggested a PSA cut-point of 0.05ng/mL or greater as a reliable PSA threshold in high-risk patients (7).

The American Urological Association and the European Association of Urology have recommended defining BCR as a serum PSA ≥0.2ng/mL followed by a second confirmatory level (16, 17). The National Comprehensive Cancer Network guidelines does not determine a specific PSA value threshold, but define BCR as rising PSA on 2 or more subsequent occasions (18).

More than fifty BCR definitions can be found in the literature (16). Although a single definition is not appropriate for all PCa patients, it is important to define an explicit recommendation that could be easily followed by clinical practitioner. We think until this moment, PSA ≥0.2ng/mL should be the standard value since it has been the most widespread BCR definition in papers and guidelines and represents a more sensitive threshold to PSA progression.

### When does it happen?

Median time to BCR ranges from 20 to 38 months (15, 19). Although BCR occurs more often in first 3 years from RP, longer follow-ups are required whereas a considerable number of patients may recur even after 15 years (20, 21). In a retrospective survey of RP performed from 1982 to 1999 with a median follow-up of 5.9 years, overall BCR (PSA ≥0.2ng/mL) rates in 5, 10 and 15 years were 16%, 28% and 39%, respectively (20). More recently, in a series comprising almost 2.500 patients submitted to RP followed longer than 10 years, authors found BCR (PSA ≥0.2ng/mL) rates of 34.3%, 44% and 52.7% at 10, 15 and 20 years after RP, respectively. Relative risk of BCR following surgery decreased with time, but late PSA increase was not negligible, as BCR rates rose by 18.4% from 10 to 20 years (21).

Many clinical and pathological variables have been shown to indicate the likelihood of BCR after RP. A single variable is not enough to predict biochemical failure. These variables should be graded into nomograms for accurately predicting BCR. In the most studied nomograms CAPRA-S and Stephenson’s nomogram, main variables significantly associated with BCR were preoperative PSA and primary and secondary Gleason grades, followed by seminal vesicle invasion (SVI), positive surgical margins (PSM), extraprostatic extension (EPE) and lymph node involvement (14, 22, 23).

Early detection is necessary to find patients with low-stage disease, while PSM is the only feature that the surgeon can influence. Thus, the 2012 US Preventive Services Task Force re-commendation to omit PSA screening from routine primary care for men concerns us, since incidence of local and regional-stage disease has reduced and distant-stage disease has increased (24). Therefore, we can expect patients that once could be cured after RP and now might experience BCR. Regarding PSM, we should be careful in the current scenario where surgeons pursue perfect functional preservation in detriment of oncological outcomes, keeping in mind the latter is the primary endpoint.

### What does it mean?

In greatest series about BCR after RP there was no man with local or distant clinical disease without an increase in PSA at the time of progression. Rising PSA is the first sign of disease progression after RP, however it does not necessarily lead men to metastatic disease or cancer mortality in few years (12, 20, 25). This is the key to understand variation among BCR definition. BCR defined as PSA ≥0.2ng/mL presents higher rates of biochemical progression, while clinical progression, assessed by metastatic disease or cancer specific mortality, was more associated with PSA ≥0.4ng/mL (14-16). Regardless the BCR definition, it shoud not be used as a landmark to start treatments. Although better oncologic outcomes were observed when salvage treatment was administrated at lower PSA levels, the correct timing of its administration depends on pathologic features, functional status, quality of life effects and patient’s preferences (26-28).

Understanding the natural history of PCa after BCR is pivotal to figure out its meaning. An excellent report of natural history of BCR was given by Johns Hopkins’s researchers. They performed a retrospective review of almost two thousand men that underwent RP for localized PCa who had not received adjuvant hormonal treatment before documentation of metastasis. Patients with good response to radiotherapy after BCR were also excluded. After a mean follow-up of 5.3 years, BCR (PSA ≥0.2ng/mL) was observed in 15% of patients at a median time of 2.3 years and 34% of them developed metastatic disease. The median actuarial time to clinically evident metastasis after PSA elevation was 8 years and it was dependent on GS 8-10, PSA-DT <10 months and time to BCR after RP. The likelyhood of metastatic disease was 37% in 5 years among those men who had presented BCR. Forty four percent of patients with metastatic disease died due to PCa. Actuarial median time to death after development of metastasis was 5 years. Death time was only influenced by time to metastatic disease after RP (25).

Han and colleagues evaluated 2.091 men submited to RP without adjuvant radiotherapy for a median follow-up of 5.9 years. Recurrence (PSA ≥0.2ng/mL) was noted in 17% of patients. Overall actuarial 5, 10 and 15 year BCR rates were 16%, 28% and 39%, respectively. Same years rates for metastatic disease were 4%, 11% and 19% and for PCa specific mortality 1%, 4% and 11%, respectively (20).

In a large and multicentric study, Eggener and colleagues used PCa pathological features to predict 15-year cancer specific mortality. In this cohort, overall 15-year PCa specific mortality rate was 7%. The only parameters significantly associated with PCa mortality were primary and secondary Gleason grades, seminal vesicle status and surgery year. Low risk patients with organ confined GS=6 cancer had disease specific mortality substantially less than the risk of death from other causes. Otherwise, PCa specific mortality in men with GS ≥8 was higher than 31% at 15 to 20 years, substantially greater than the risk of death from competing causes. Increased risk of PCa mortality was also observed among men with SVI and lymph node metastasis. Year of surgery was a relevant risk factor with an improved prognosis in contemporary patients. Positive surgical margins and EPE were not significantly associated with PCa mortality (29).

Short time from RP to BCR has also been associated with poor clinical prognosis. Ten-year rate of PCa systemic progression ranges from 10% to 19% for patients who experienced BCR >6 years or <1.2 year after RP, respectively. Although the time interval from RP to BCR is not an independent variable related to prognosis, patients with early BCR have higher GS, greater preoperative PSA levels, faster PSA-DT and more advanced tumor stage, factors associated to systemic progression and PCa mortality (19).

A critical issue in the management of BCR after RP is determining wheter a rising PSA reflects local or distant recurrence. The most important factors that predict distant recurrence are pathologic GS, pathologic tumor stage and short PSA-DT (19). At low PSA levels that determine BCR, few imaging studies are under investigation to distinguish the sort of recurrence. Preliminary studies suggest that performing Ga-prostate-specific membrane antigen (PSMA) positron emission tomography/computed tomography (PET/CT) in patients with rising PSA can be useful for re-staging PCa, even changing treatment strategies (8). While new technologies have been incorporated in BCR management, stratifying patients into risk groups is still crutial, as it can determine different prognosis and treatments.

### Differences among risk groups

Men with PCa have been classified into low-, intermediate- and high-risk Groups for tumor recurrence and disease specific mortality, based on PSA level, clinical or pathological staging and GS. High-risk patients have PSA level ≥20ng/mL or GS ≥8 or clinical/pathological stage ≥T2c (30). Lymph-node positive (pN1) and PSM have also been reported as poor prognosis factors.

Risk Group classification predicts biochemical and clinical progression as well as PCa specific mortality and overall survival. The risk of disease progression in these groups has been validated for patients submited to RP in many studies. In patients from Mayo Clinic, BCR rates were 2.3 and 3.3-fold greater in high and intermediate-risk in comparison with low-risk patients, respectively. In those patients, mortality rates in high and intermediate-risk patients were greater than 11 and 6-fold over low-risk men (31).

Therefore, it is crutial to understand the role of each clinical and pathologic feature in PCa BCR and disease progression.

### Pre-operative Prostate Specific Antigen (PSA) and PSA doubling-time (PSA-DT)

PSA is the main biochemical tool for diagnosing and following patients with PCa. Men with preoperative PSA ≥20ng/mL present a BCR risk 3.7-fold greater than those men with PSA <6ng/mL (14, 22, 23). PSA-DT is also a good predictor of distant recurrence and PCa specific mortality after RP. Systemic progression and death from PCa are almost 5-fold more often in patients with PSA-DT <6 months if compared with PSA-DT >10 years (19). However, its clinical usefulness is limited since a period of 6 months to 2 years of BCR is required for accurate calculation and sometimes treatment decisions should be taken earlier (29).

### Gleason score

Primary and secondary Gleason grades are associated with higher BCR rates and are significant predictors of metastatic disease and PCa specific mortality (12, 19). Gleason score ≥8 is associated to BCR 3.3-fold higher than GS=6 (22, 23). The 15-year PCa mortality rates for pathological GS=6, 3+4, 4+3 and 8-10 could be as great as 1,2%, 6,5%, 11% and 37%, respectively. Men with GS=6 have negligible risk of cancer death, in contrast with men with intermediate and high-risk PCa (29).

### Extraprostatic extension (EPE)

Extraprostatic extension (pT3a or greater) appears to influence BCR, but it is not an independent factor for cancer specific mortality (22, 23, 29). EPE increases BCR risk 1.5-fold over confined disease. The impact of the degree of EPE has been assessed for various methods, including Epstein’s criteria and Wheeler method (32, 33). Extension of EPE has been shown as an independent predictor of BCR in pT3a prostate cancer, even without PSM. However, the diference between focal and non-focal EPE has no impact in PCa specific mortality or overall survival (34, 35).

### Seminal vesical invasion (SVI)

Invasion of the seminal vesicles (pT3b) after RP has historically been associated with a poor prognosis. SVI increases BCR risk in 2.3-fold and PCa specific mortality rates are 22% highwer (22, 23, 29). It suggests a poorer prognosis in men with pT3b disease in comparisson with pT3a. Although the majority of these men have high-grade disease and worse outcomes, some authors have found a subset of patients with SVI with favorable prognosis. Isolated SVI, with negative surgical margins, negative lymph nodes, lower GS and PSA represents a small portion of men with PCa that has better prognosis (36, 37).

### Positive surgical margins (PSM)

PSM alone is not associated with higher risk of PCa mortality, although it increases men’s risk of BCR in 2 to 4-fold and need for secondary therapy (38). Some authors have attempted to stratify the number and extent of PSM. Although greater number and extension of PSM are associated with an increased risk of BCR compared to solitary and focal PSMs, its clinical usefullness is limited (39). PSM location is also associated to different BCR rates. Bladder neck margin involvement seems to present greater risk of BCR than other sites (40).

### Lymph-node positive

Patients with positive node disease have recurrence rates 1.7-fold greater than negative node disease. Lymph node metastasis can increase PCa specific mortality in more than 40% in long-term follow-up (29). Time to progression is significantly correlated with the number of diseased nodes. Biochemical and clinical relapse time can range from 7 to 28 months and from 24 to 46 months, if we consider patients with one or greater than 2 positive nodes, respectively (41). Median PCa specific survival in those men can be as great as 12 years. Men with minimal metastatic nodes undergoing RP with meticulous pelvic lymph node dissection may remain free of BCR longer than 10 years (42).

## CONCLUSIONS

A non-negligible amount of patient presents BCR after RP. Understanding BCR meaning and providing a single definition will help us to standardize and to compare outcomes among studies. Despite the number of BCR definitions, PSA ≥0.2ng/dL is the most used one. PSA relapse help us to guide clinical practice, but it should not be the only feature to determine the therapeutic approach.

There are many variables related to BCR such as GS, pathological stage, surgical margin and lymph-node status. These variables have to be considered together, as they help us to predict local or distant recurrence. Short PSA-DT (mainly PSA-DT <6 months), GS ≥8, SVI (pT3b) and lymph-node positive appears to be the main factors associated to metastatic disease and PCa mortality. With this in mind, stratifying men with PCa into risk groups is pivotal to define prognosis and treatment. This review provides us an updated assessment of BCR definition and meaning, showing us the differences of BCR rates and clinical outcomes among risk Groups.
